# Supplementary material for: Insight into Calcium-Binding Motifs of Intrinsically Disordered Proteins
Source: Biomolecules. 2021 Aug 9;11(8):1173. doi: 10.3390/biom11081173 (PMC8391695; doi:10.3390/biom11081173)
Supplement: Supplementary file 1 [file biomolecules-11-01173-s001.zip › biomolecules-1313272-supplementary.pdf]

biomolecules

SUPPORTING FIGURES AND TABLES

Insight into calcium-binding motifs of intrinsically disordered proteins

Estella A. Newcombe [1,3], Catarina B. Fernandes [1,2], Jeppe E. Lundsgaard [1,2], Inna Brakti [1,2], Kresten Lindorff-Larsen [1], Annette E. Langkilde [3], Karen Skriver [1,2], and Birthe B. Kragelund [1,2*]

SUPPORTING TABLES

Table S1 – Protein sequences

Table S2 – Protein purification and production methodology

SUPPORTING FIGURES

Figure S1 – ANAC046$_{172-338}$ secondary chemical shifts.

Figure S2 – Amide HSQCs for proteins in the presence and absence of calcium chloride

Figure S3 – Scatchard plots for proteins and SM1-4 peptides, measured via OCPC assay

Figure S4 – SM1-4 peptide TOCSY, ROESY, and amide HSQCs spectra with assignments in the presence and absence of calcium chloride

Table S1 – Protein Sequences

Protein	Sequence
aSN_{96-140}	KKDQL GKNEEGAPQE GILEDMPVDP DNEAYEMPSE EGYQDYEPEA
aSN_{1-140}	MDVFMKGLSK AKEGVVAAAE KTKQGVAEAA GKTKEGVLYV GSKTKEGVVH GVATVAEKTK EQVTNVGGAV VTGVTAVAQK TVEGAGSIAA ATGFVKKDQL GKNEEGAPQE GILEDMPVDP DNEAYEMPSE EGYQDYEPEA
$ANAC046_{172-338}$	NAPSTTITT TKQLSRIDSL DNIDHLLDFS SLPPLIDPGF LGQPGPSFSG ARQQHDLKPV LHHPTTAPVD NTYLPTQALN FPYHSVHNSG SDFGYGAGSG NNNKGMIKLE HSLVSVSQET GLSSDVNTTA TPEISSYPMM MNPAMMDGSK SACDGLDDLI FWEDLYTS
$NHE1_{680-815}$	I NNYLTVPAHK LDSPTMSRAR IGSDPLAYEP KEDLPVITID PASPQSPESV DLVNEELKGK VLGLSRDPAK VAEEDEDDDG GIMMRSKETS SPGTDDVFTP APSDSPSSQR IQRCLSDPGP HPEPGEGEPF FPKGQ
DSS1wt	MSRAALPSLE NLEDDDEFED FATENWPMKD TELDTGDDTL WENNWDDEDI GDDDFSVQLQ AELKKKGVAA C
ProTα	MSDAAVDTSS EITTKDLKEK KEVVEEAENG RDAPANGNAE NEENGEQEAD NEVDEEEEEG GEEEEEEEEG DGEEEDGDED EEAESATGKR AAEDDEDDDV DTKKQKTDED D

Table S2 – Protein Purification and Production Methodology

**Protein**	**IPTG (mM)**	**Lysis/binding buffer**	**Wash buffer**	**Elution buffer**	**Cleavage buffer**
aSN_{96-140}	1	50 mM Tris (pH 8.0), 150 mM NaCl, 10 mM Imidazole	50 mM Tris (pH 8.0), 1 M NaCl, 10 mM Imidazole	50 mM Tris (pH 8.0), 150 mM NaCl, 250 mM Imidazole	50 mM Tris (pH 8.0), 150 mM NaCl
$ANAC046_{172-338}$	1	20 mM NaH_2PO_4 pH 7.0, 500 mM NaCl, 5 mM Imidazole	20 mM NaH_2PO_4 pH 7.0, 500 mM NaCl, 20 mM Imidazole	20 mM NaH_2PO_4 pH 7.0, 500 mM NaCl, 300 mM Imidazole	20 mM NaH_2PO_4 pH 7.0, 1 mM DTT
DSS1wt, swap, E, D	0.1	50 mM Tris (pH 8.0), 150 mM NaCl, 10 mM Imidazole	50 mM Tris (pH 8.0), 1 M NaCl, 10 mM Imidazole, 1mM β-mercaptoethanol	50 mM Tris (pH 8.0), 1 M NaCl, 250 mM Imidazole, 1mM β-mercaptoethanol	50 mM Tris (pH 8.0), 150 mM NaCl, 1 mM DTT

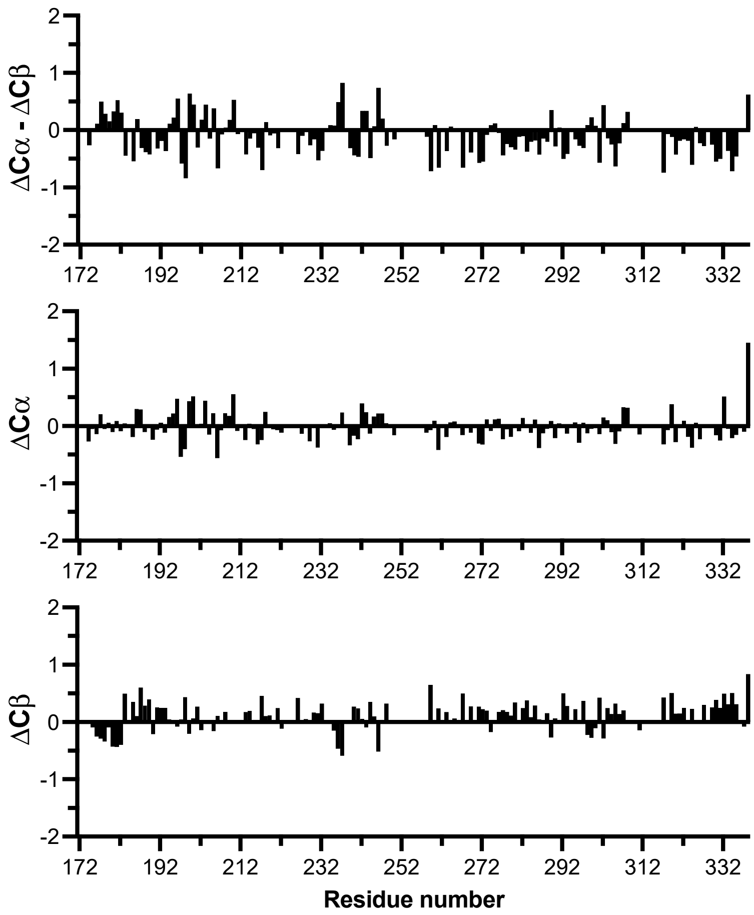

Figure S1. ANAC046$_{172-338}$ secondary chemical shifts. Calculated using random coil chemical shifts for IDPs [65-67].

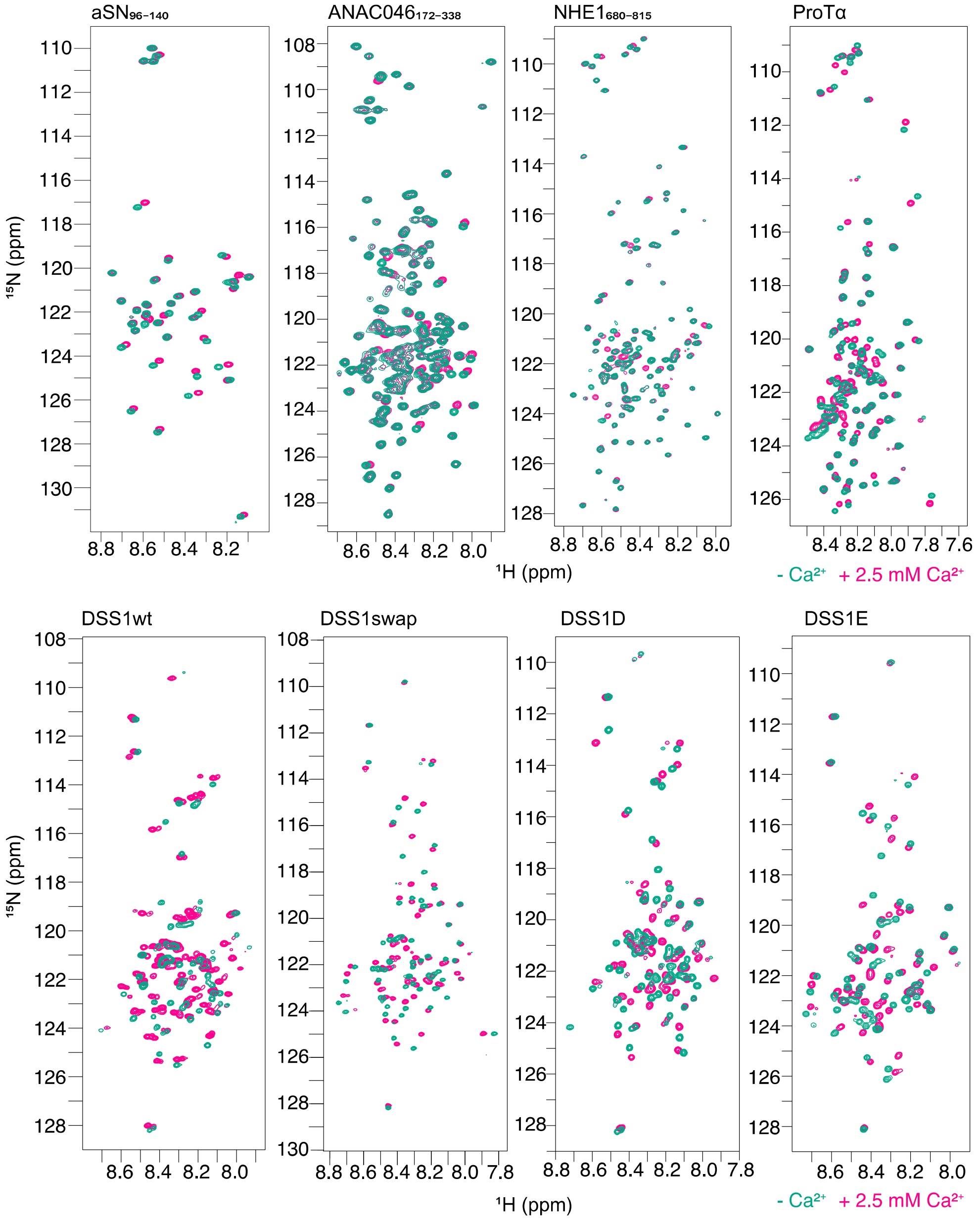

Figure S2. Amide HSQC spectra of all proteins tested, with (pink) and without (teal) calcium. Assignments can be found using the following BMRB accession numbers: ANAC046$_{172\text{-}338}$: 51033; NHE1$_{680\text{-}815}$: 27812; ProTα: 27215; DSS1: 27618. aSN assignment as described previously [28].

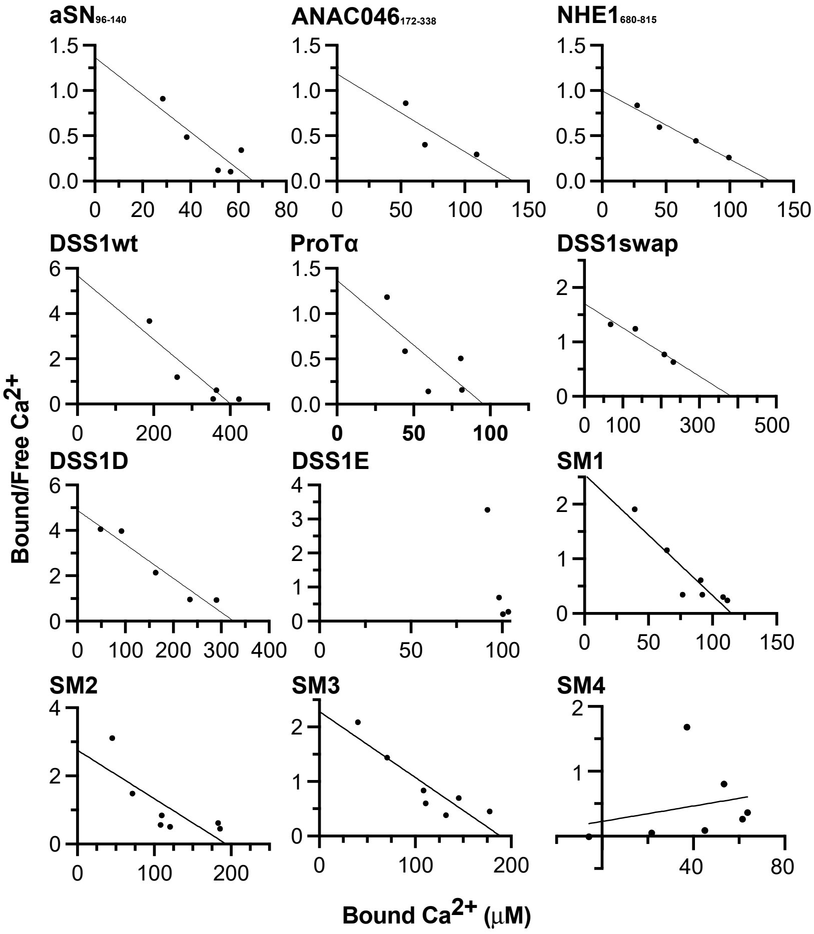

Figure S3. Scatchard plots of proteins (100 μM; ProTα: 10 μM) and SM1-4 (200 μM) with extrapolated linear regression. Buffer conditions for OCPC assay: Tris-HCl (15 mM) pH 8.0.

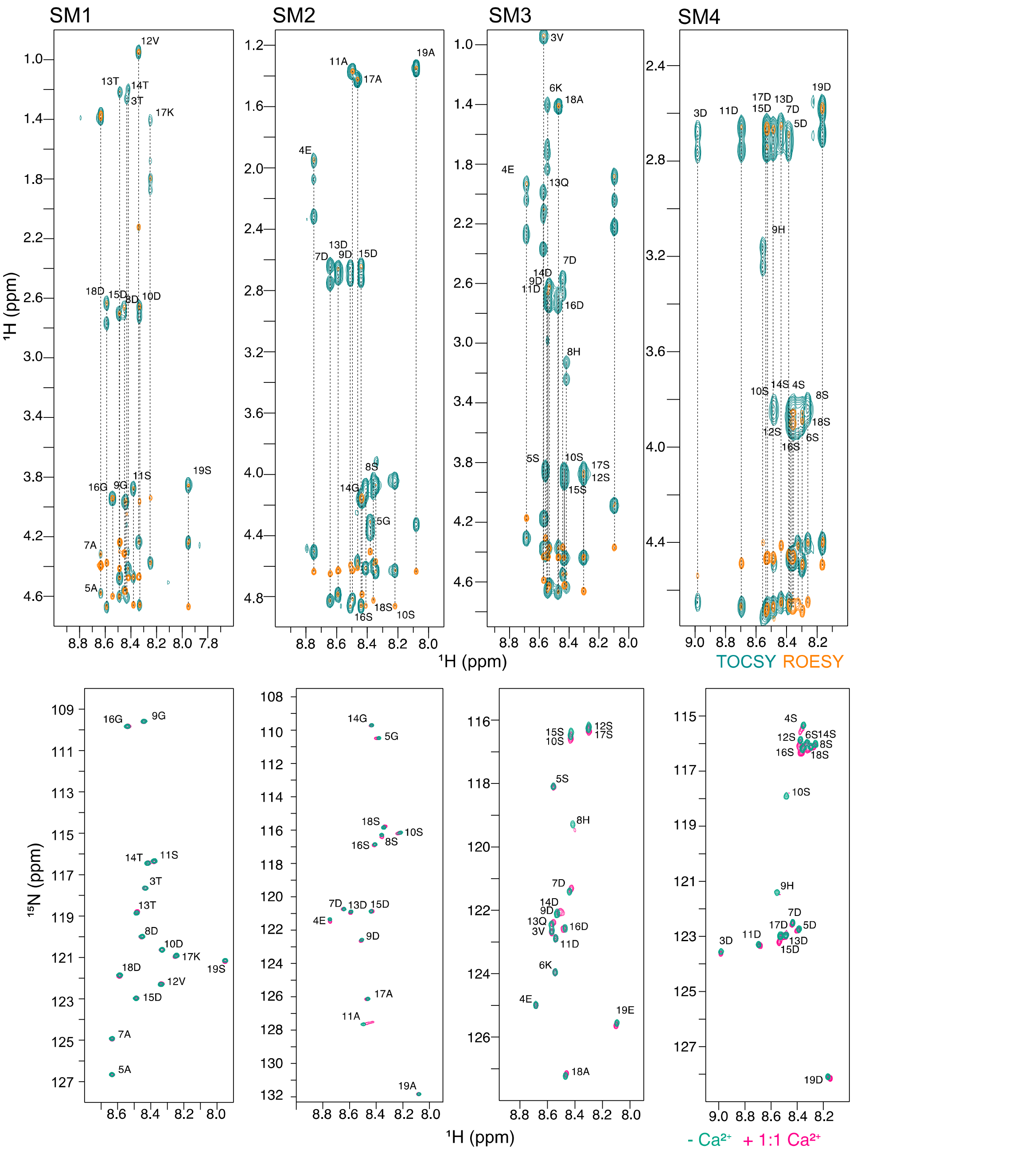

Figure S4. TOCSY (teal) and ROESY (orange) fingerprint regions for SM1-4, as used for peptide assignment (upper panels). Amide HSQC spectra of SM1-4, with (pink) and without (teal) calcium (lower panels).

Reference:

65. Kjaergaard, M.; Poulsen, F.M. Sequence correction of random coil chemical shifts: Correlation between neighbor correction factors and changes in the Ramachandran distribution. *J. Biomol. NMR* **2011**, *50*, 157–165, doi:10.1007/s10858-011-9508-2.
66. Kjaergaard, M.; Brander, S.; Poulsen, F.M. Random coil chemical shift for intrinsically disordered proteins: Effects of temperature and pH. *J. Biomol. NMR* **2011**, *49*, 139–149, doi:10.1007/s10858-011-9472- x.
67. Schwarzinger, S.; Kroon, G.J.A.; Foss, T.R.; Chung, J.; Wright, P.E.; Dyson, H.J. Sequence-dependent correction of random coil NMR chemical shifts. *J. Am. Chem. Soc.* **2001**, *123*, 2970–2978, doi:10.1021/ja003760i.
